# 

**Published:** 1903-07-11

**Authors:** 


### NOTICE TO CORRESPONDENTS.

All MSS., Letters, Books for Review, and other matters intended for the Editor should be addressed The Editor, "The Hospital," The Hospital Buildings, 28 & 29 Southampton Street, Strand, W.C.

The Editor cannot undertake to return rejected MSS., even when accompanied by stamped directed envelope.

**Notice to Advertisers and Subscribers.**

All Advertisements, Orders for Copies of The Hospital, and Business Communications should be addressed to **THE MANAGER, (Not to the Editor),** The Hospital, 28 & 29 Southampton Street, Strand, London, W.C.

The Cost of Subscription, post free, is as follows.—Per week, 3½d.; per quarter, 3s. 9d.; per half-year, 7s. 6d.; per annum, 15s. Foreign and Colonial:—Per annum, 19s. 6d., payable, in all cases, in advance.

**NOTICE.—Vol. XXXIII. of "The Hospital" (October 1902 to March 1903) in handsome cloth binding, will shortly be on sale at the office of this Journal, price 7s. 6d. Cases for binding the Numbers contained in this Volume 1s. 6d. Index to Vol. XXXIII., 2½d., post free.**

**The monthly part for July is now ready. Price 1s., by post 1s. 3d.**

### The Student of Medicine.

**APPOINTMENTS.**

**John Brangan**, L.R.C.P., L.R.C.S.Edin., L.F.P.S.Glas., Coroner for North Meath. **W. Crerar**, M.B.Glas., District Medical Officer of the Rotherham Union. **J. F. Dwyer**, L.R.C.P., L.R.C.S.Edin., District Medical Officer of the Crickhowell Union. **Ernest W. Hey Groves**, M.D., B.Sc.Lond., M.R.C.S., L.R.C.P., Assistant Surgeon to the Bristol General Hospital. **R. L. Guthrie**, M.A., M.D., Barrister-at-Law, Deputy Coroner for the North-Eastern District of the County of London. **H. G. Kyle**, B.A., M.B., B.Ch.Oxon., Surgeon to the Bristol General Hospital. **Thomas A. Mendes**, L.R.C.P.&S.Edin., L.F.P.&S Glas., Second Assistant Medical Officer to the County and City Asylum, Hereford. **T. W. S. Morgan**, M.R.C.S.Eng., L.S.A., District Medical Officer to the Clutton Union. **Catherine Mary Richardson**, M.B., Ch.B.Edin., House-Surgeon to the Derbyshire Hospital for Sick Children, Derby. **Charles Roberts**, F.R.C.S., Surgical Registrar to the Manchester Royal Infirmary. **Professor Antony Roche**, M.R.C.P., L.R.C.S.Irel., Lecturer on Public Health to Maquorn College. **Thos. Rowell**, M.B., B.S.Durh., Second Assistant Medical Officer at the City Asylum, Newcastle-on-Tyne. **T. O'D. Russell**, M.R.C.P., F.R.C.S.Irel., District Medical Officer of the Hexham Union. **J. E. H. Sawyer**, M.A., M.D.Oxon., Honorary Administrator of Anæsthetics to the Birmingham Dental Hospital. **R. G. Selby**, M.B., C.M.Edin., District Medical Officer of the Rotherham Union. **H. Hamilton Serpell**, M.R.C.S., L.R.C.P., Assistant House-Surgeon to the South Devon and East Cornwall Hospital at Plymouth. **J. H. Shepherd**, M.B., Ch.B., Second Assistant to Dundee Royal Lunatic Asylum. **C. W. Smeeton**, M.R.C.S., L.R.C.P.Lond., District Medical Officer of the Malton Out-relief Union. **Septimus Sunderland**, M.D., M.R.C.P., Obstetric Physician to the French Hospital, Shaftesbury Avenue. **E. D. Telford**, F.R.C.S., Resident Surgical Officer to the Manchester Royal Infirmary. **W. J. Woodyatt**, M.B., B.S.Vict., Assistant Medical Officer of the Whitechapel Union.

**"The Hospital" Institutional Library.**

"Hospitals and Asylums of the World." 4 Vols., with a Portfolio of Plans, complete £12 12s.

"Burdett's Hospitals and Charities, 1902." 5s.

"Burdett's Official Nursing Directory, 1903." 3s. net.

"Cottage Hospitals, General, Fever, and Convalescent." 10s. 6d.

"Uniform System of Accounts for Hospitals and Public Institutions." New Edition. 4s. net.

"Hospital Expenditure: The Commissariat." 2s. 6d.

"A Legal Handbook for the Use of Hospital Authorities." 2s. 6d.

"The Cottage Hospital Case Book or Register of Patients." 10s. and 12s. 6d.

"The Furnishing and Appliances of a Cottage." 6d.

All these are published by the Scientific Press, Ltd., and may be obtained through any bookseller, or direct from the publishers, 28 and 29 Southampton Street, Strand, London, W.C.

### Books on the Care of the Young.

### The Physiological Feeding of Infants.

By Dr. ERIC PRITCHARD.

Price **1/-** post free.

A most useful guide to artificial feeding.

### The Mother's Help and Guide to the Domestic Management of her Children.

By Dr. MURRAY BRAIDWOOD.

Price **2/-** post free.

Useful in health and illness.

### Cleanliness in Children.

By ROSE PETTY.

Price **2d.** post free.

Will aid the School Nurse in her mission.

### Sight and Hearing in Childhood.

By R. BRUDENELL CARTER, F.R.C.S., and ARTHUR CHEATLE, F.R.C.S.

Price [illegible]/- net, post free **2/3.**

This is a most useful and important work by two eminent authorities.

### Spinal Curvature and Awkward Deportment.

By Dr. GEORGE MÜLLER.

Price **2/6** post free.

This is a book which should be in the hands of all who have the care of children, as it points out both prevention and cure in simple language.

### Some Health Aspects of Education.

By Dr. PERCY LEWIS.

Price **1/-** post free.

### Home Nursing of Sick Children.

By J. D. E. MORTIMER, F.R.C.S.

Price **1/-** post free.

A most useful little book, full of practical hints on a difficult subject.

*These books are obtainable of all Booksellers, and are published by* THE SCIENTIFIC PRESS, LIMITED, 28 & 29 Southampton Street, Strand, London, *who will send their latest Catalogue of Nursing Text-books post free to any Nurse on application.*

